# Effects of walking football on adherence, safety, quality of life and physical fitness in patients with prostate cancer: Findings from the PROSTATA_MOVE randomized controlled trial

**DOI:** 10.3389/fonc.2023.1129028

**Published:** 2023-03-21

**Authors:** Andreia Capela, Pedro Antunes, César André Coelho, Catarina Laranjeiro Garcia, Sandra Custódio, Rui Amorim, Telma Costa, Eduardo Vilela, Madalena Teixeira, Anabela Amarelo, Joana Silva, Ana Joaquim, Sofia Viamonte, João Brito, Alberto J. Alves

**Affiliations:** ^1^ ONCOMOVE® – Associação de Investigação de Cuidados de Suporte em Oncologia (AICSO), Vila Nova de Gaia, Portugal; ^2^ Centro Hospitalar Vila Nova de Gaia – Espinho, Entidade pública empresaríal (EPE), Vila Nova de Gaia, Portugal; ^3^ Research Center in Sport Sciences, Health and Human Development (CIDESD), Sport Sciences Department, University of Beira Interior, Covilhã, Portugal; ^4^ Research Center in Sport Sciences, Health and Human Development (CIDESD), Physical Education and Sport Sciences Department, University of Maia, Maia, Portugal; ^5^ Portugal Football School, Federação Portuguesa de Futebol, Oeiras, Portugal

**Keywords:** walking football, adherence, safety, quality of life, physical fitness, prostate cancer, rct

## Abstract

**Aims:**

To analyze the feasibility and impact of a walking football (WF) program on quality of life (QoL), cardiorespiratory fitness (CRF), muscle strength, and balance program in men with prostate cancer under androgen deprivation therapy (ADT).

**Methods:**

Fifty patients with prostate cancer (stages IIb-IVb) under ADT were randomized to a 16-week WF program plus usual care (n=25) or usual care control group (n=25). The WF program consisted of three 90-minute sessions per week. Recruitment, withdrawal, adherence, enjoyment rate, and safety of the intervention were recorded throughout the study. Cardiorespiratory fitness was assessed before and after the interventions, while handgrip strength, lower limb muscle strength, static balance, and QoL were assessed before, during (week 8), and after (week 16) the interventions. Adverse events during sessions were also recorded.

**Results:**

The WF group showed high levels of adherence (81.6 ± 15.9%) and enjoyment rate (4.5 ± 0.5 out of 5 points). In the intention-to-treat analysis, the WF group showed an improvement in chair sit-to-stand (p=0.035) compared to the control group. Within-group comparisons showed that handgrip strength in the dominant upper limb (p=0.024), maximal isometric muscle strength in the non-dominant lower limb (p=0.006), and balance in the dominant limb (p=0.009) improved over time in the WF group but not in the usual care group. The results obtained from the per-protocol analysis indicate that CRF improved significantly in the WF group as compared to the control group (*p*=0.035). Within-group analysis revealed that CRF (*p*=0.036), muscle strength in dominant (*p*=0.006) and non-dominant (*p*=0.001) lower limbs, and balance in the non-dominant lower limb (*p*=0.023) improved after 16 weeks of WF, but not in the control group. One major traumatic injury (muscle tear) was reported with a complete recovery before the end of the intervention.

**Conclusion:**

This study suggests that WF is feasible, safe, and enjoyable in patients with prostate cancer under hormonal therapy. Furthermore, patients who adhere to the WF program can expect cardiorespiratory fitness, muscle strength, and balance improvements.

**Clinical trials registration:**

clinicaltrials.gov, identifier NCT04062162.

## Introduction

Prostate cancer is the second most frequent cancer and the fifth leading cause of death from cancer in men worldwide (1). With the purpose of delaying disease progression and enhancing survival, ADT is widely used as a stand-alone treatment or in conjunction with radiation therapy or radical prostatectomy (2). However, despite its undeniable clinical importance, the use of ADT is associated with a vast spectrum of potential side effects (namely loss of muscle mass, bone mass, and physical functionality, increases in fat mass, fatigue, worse metabolic, glycemic, and cardiovascular profile) that considerably reduce QoL. Importantly, an increasing number of patients might be on ADT for prolonged periods and might survive several years following the cessation of the treatments (3). Therefore, it is crucial to implement preventive strategies that contribute to mitigating the toxicity of ADT (4).

Exercise has been proposed as a non-pharmacological useful and viable strategy to counteract some adverse effects of androgen deprivation therapy (5). Exercise has been included in the clinical guidelines from the European Society of Medical Oncology (4), European Association of Urology (6), and the American Society of Clinical Oncology (7). To date, most randomized controlled trials evaluating exercise programs in patients under ADT comprised structured supervised or home-based interventions that commonly combined traditional aerobic (such as walking, jogging, or bicycling) and strength training (8). Despite promising results, such programs may be inadequate to engage and maintain men with prostate cancer in long-term interventions (9). Moreover, permanent behavioral changes concerning engagement with regular physical activity might be difficult to implement in a real-world setting. Indeed, recent data suggest that men with prostate cancer prefer to exercise in a structured group environment, which appears to facilitate the uptake of exercise programs and enhance long-term adherence in this patient population (10). Therefore, developing novel interventions that combine patients’ needs, characteristics, and preferences is important.

The popularity of football worldwide, especially among men, appeals to its potential as a health-enhancing recreational physical activity. Currently, several studies on patients with prostate cancer provide interesting results about the multiple beneficial effects of recreational football-based interventions on distinct health outcomes (11, 12), and it is well-established that playing recreational football can also promote enjoyment and positive effects on mental and social well-being (13). However, given its intermittent nature, vigorous efforts, and the possible risk of injuries (due to the potential contact between participants, duels, and tackles), clinicians might be cautious about recommending recreational football practice in patients with prostate cancer undergoing ADT. Adverse events associated with recreational football practice have been reported and might constitute a relevant barrier to the implementation of such programs in these patients (14, 15), who are typically characterized by advanced age, low physical activity levels, and poor fitness (16). To try to minimize potential risks, injuries, and side effects, an adapted version of football has emerged over recent years. Walking football (WF) adheres to the general rules of football, but participants are not allowed to run or engage in physical contact with each other (17). Studies showed that WF programs generally presented high levels of adherence and enjoyment (18–21), and the low rate of adverse events described suggests that it is a feasible and safe exercise strategy (22). In the advanced prostate cancer population, bone metastasis (23) and osteoporosis (6, 24) can be a major concern in the implementation of recreational football practices.

The intensity of WF training characterizes it as generally a light-to-vigorous physical activity (22), which led to promising results on body composition, aerobic fitness, and blood pressure in middle-aged and older individuals (21, 25). However, the effectiveness of WF practice has not been tested in men with prostate cancer undergoing ADT. Given this background, the main aim of this study was to analyze the quality of life and feasibility of a WF program in men with prostate cancer undergoing ADT. The secondary aim was to measure the impact of WF practice on CRF, muscle strength, and balance.

## Methods

### Study design

This study was a prospective randomized clinical trial, with a parallel 2-arm group design. Patients were recruited by physicians of the Oncology and Urology departments of the Vila Nova de Gaia-Espinho Hospital Centre, Portugal. Patients were randomly allocated to a 16-week WF program plus usual care (intervention group) or usual care alone (control group). Primary and secondary outcomes were assessed at baseline, after 8 weeks of intervention, and 2 days after 16 weeks of intervention, except for CRF, which was assessed only at baseline and after the 16-week intervention. All patients provided written informed consent. The study was approved by the hospital ethics committee (50/2019-2) and registered in clinicaltrials.gov (NCT04062162).

### Participants

Adult patients with prostate cancer undergoing ADT for at least 6 months were enrolled in the study if they presented the following inclusion criteria (1): patients treated with radical prostatectomy more than one month passed the procedure and with approval from the urologist (2); patients previously treated with prostatic radiotherapy, at least one month after the end of radiotherapy treatment and with approval from the oncologist; (3) adult patients undergoing hormone therapy with a luteinizing hormone-releasing hormone (LHRH) analogue or antagonist as an initial approach or in the setting of biochemical recurrence. Exclusion criteria included osteoporosis (spine or femur T score of -2.5 or lower) and contraindications for exercise training such as acute coronary syndromes, acute endocarditis, myocarditis or pericarditis, decompensated heart failure, severe aortic stenosis, uncontrolled arrhythmia, uncontrolled hypertension, or any physical disability that precludes safe and adequate exercise testing and training according to the attending physician’s assessment (26). All participants were evaluated by a rehabilitation medicine specialist before study entry.

### Randomization and allocation

Permuted block randomization was generated with balanced groups (1:1), and strata were defined by age (lower and greater than 65 years) using electronic software (www.sealedenvelope.com).

### Outcomes

The primary outcomes were QoL and feasibility assessed by the recruitment rate (the number of invited patients divided by the number of those enrolled), acceptability (number of patient withdrawals and dropouts), adherence (number of sessions attended, number of sessions missed and level of enjoyment) and retention (the number of patients who completed all the exercise sessions divided by the number of patients allocated to the exercise group) of the WF program. The level of enjoyment with the WF program was assessed by a Likert scale (1-not at all satisfied to 5-totally satisfied). Secondary outcomes included CRF, muscle strength, balance, and adverse effects during/after the exercise sessions (e.g., falls and injuries).

### Procedures

#### Clinical and demographic data

Socio-demographic and clinic-pathologic data were collected through patient clinical records.

#### Quality of life

QoL was assessed using the European Organization for Research and Treatment of Cancer (EORTC) quality of life scale – QLQ30, and its specific module for prostate cancer – PR25 (27).

#### Cardiorespiratory fitness

CRF was assessed at baseline and after 16 weeks of intervention through a symptom-limited treadmill exercise stress test on a treadmill using a Bruce protocol, and metabolic equivalents (METs) were calculated according to the stage of protocol and time reached at peak exercise. The maximum heart rate (HR) achieved was also recorded for the determination of the intensity of exercise sessions.

#### Muscle Strength

Maximum voluntary handgrip strength was measured using a digital hand dynamometer (Saehan model SH1001, DHD-1, Saehan Corp. South Korea). Each participant performed a total of 6 trials, 3 on each hand, with an alternating bilateral sequence. Before each trial, the position of the limb was adjusted so that each participant placed the elbow flexed at a 90° angle with the wrist as close to 0° as possible. The average of the respective tests on each member was determined for analysis.

Maximum isometric muscle strength of the knee extensors was measured on both limbs with a digital dynamometer (Advanced Force Gauge, 2500N, Mecmesin Limited, Slinfold, West Sussex, United Kingdom). The participant remained seated during the test with the lower limb flexed at 90°. Two repetitions were performed on each limb and the average value was recorded.

The 30-second chair sit-to-stand test was also used to evaluate muscle strength and endurance of the lower limbs (28). Each participant was instructed to stand up and sit as many times as possible on a 40-cm-high chair for 30 seconds, keeping arms crossed close to the chest (28). The result was determined by the number of repetitions.

#### Balance

The single-leg stance test with eyes open was used to assess static balance in the dominant and non-dominant limbs. Each participant remained with their arms crossed over their chests and supported in one leg for as long as possible. Time recording began when the patient raised the foot from the floor and ended when the patient either (1): uncrossed his arms, (2) moved the raised foot or touched the floor, (3) moved the weight-bearing foot, and (4) reached the maximum 45-second time (29). An average of 3 trials were recorded for each limb.

#### Safety

Adverse effects (AEs) during WF practice were recorded and classified according to the consensus defined by Fuller et al. (30), and their severity was graded. Data on location, type, body side, mechanism of injury (traumatic or overuse), recurrence, time of intervention, the context of the injury (e.g., contact with another participant or object), breach of protocol rules, time until reintegration into an exercise routine, number of missed sessions, need for medical evaluation, date, and description of circumstances of occurrence) were recorded.

### Study intervention

The exercise intervention consisted of 3 weekly sessions of WF, on non-consecutive days, for a period of 16 weeks (a total of 48 sessions). The exercise sessions took place at an indoor sports hall, and were divided into four sequential phases (1): a warm-up phase that involved joint mobility exercises and balance exercises (15 min); (2) a skill-developing phase where patients developed football-specific technical skills, such as passing, dribbling, and shooting, as well as fundamental motor skills, including aerobic power, muscular endurance and balance (50 min); (3) a structured small sided game (e.g., 7 vs. 7 or 5 vs. 5) of WF (20 min); and (4) a cool-down phase (5 min). The training sessions were designed, planned, and supervised by a certified football coach (UEFA B license) and two exercise physiologists.

Exercise intensity was continuously monitored during sessions with HR monitors (Firstbeat Sports, Firstbeat Sport^®^, Finland). Maximum HR was recorded during baseline maximal exercise testing to calculate the intensity of exercise sessions. Effort during exercise sessions was controlled by the rating of perceived exertion (RPE) through the Borg 6-20 scale (minimum effort = 6; maximum effort = 20). Participants were encouraged to exercise with moderate-to-vigorous intensity, as recommended for adults and older adults (64-76% to 77-95% of maximum HR, reporting 12-17 [“a little difficult” to “very difficult”] Borg 6-20 scale) (31). The amount of time spent in very light (1-56%), light (57-63%), moderate (64-76%), vigorous (77-95%), and maximum exercise intensity (96-100%) was determined based on maximum HR, obtained during the treadmill exercise stress test, according to the American College of Sports Medicine (ACSM) physical activity recommendations for adults (31). The control group had only usual medical care, which involves routine follow-up appointments with the attending physician, regular assessments of blood count and bone mineral density, as well as general counseling on issues related to physical inactivity and weight gain. In patients with metastatic prostate cancer, usual care additionally encompasses bone scintigraphy and positron emission tomography/computed tomography (PET/CT) assessments. However, there was no provision for physical activity support as part of the usual care. This group was offered the opportunity of joining the WF program after the 16-week study period. However, although patients of the control group were enrolled later in the WF program, their participation had to be cancelled due to the start of the COVID-19 pandemic.

### Statistical analysis

Exploratory data analysis and Shapiro-Wilk tests were performed to determine the normality of the data distribution. Continuous variables are expressed as mean (SD) or median (interquartile range), whereas for categorical variables, counts and percentages are presented. Between-group differences at baseline were tested with unpaired student-t tests or chi-square tests. Two-factor mixed ANOVA was used to assess the effect of the intervention over time across groups in variables with normal distribution and paired-sample ANOVA was performed for within-group comparisons from baseline to the end of the study. Friedman and Wilcoxon’s tests were used for within-group comparisons in variables with no normal distribution. Furthermore, we performed a per-protocol analysis including only patients with adherence of 70% or greater to the scheduled exercise sessions. All analyses were conducted with SPSS version 24.0 (SPSS Inc., Chicago, IL, USA). The level of significance was set as P < 0.05.

## Results

### Participants

**Figure 1:**
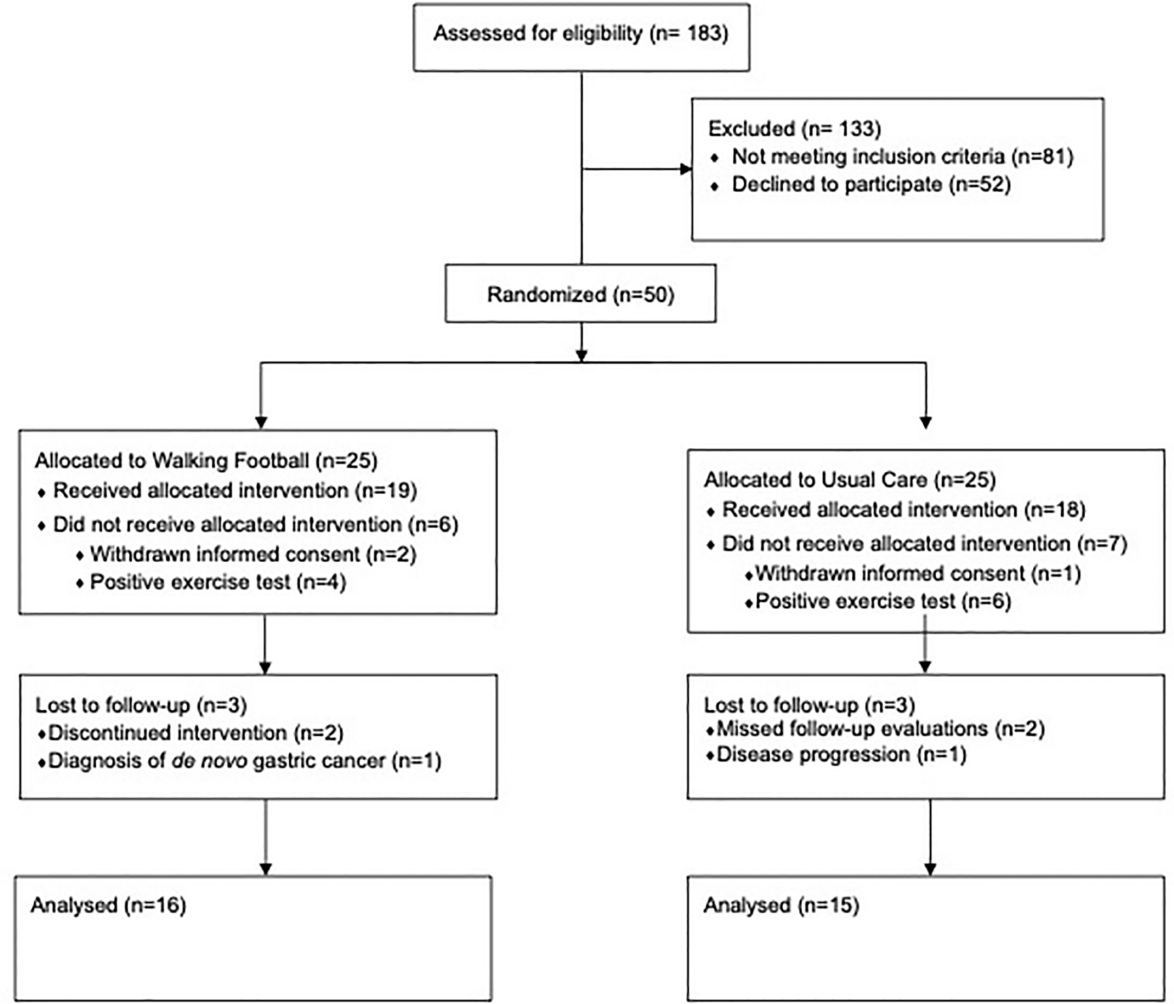
Flow diagram depicting the study design.

Of the 50 patients who were considered eligible to participate in the study (**Figure 1**), 3 refused to participate and 10 were excluded due to electrocardiographic changes during exercise testing. In addition, 2 patients in the exercise group discontinued the intervention and 1 was excluded due to a *de novo* gastric cancer diagnosis. Also, 2 patients in the control group missed follow-up assessments and 1 patient had disease progression. In total, 31 patients were included in the analysis, 16 in the WF group and 15 in the control group. The patient’s characteristics are shown in **Table 1**. Patients were mostly older adults (71.8 ± 5.9 years) with excess body weight (Body Mass Index: 28.3 ± 4.1 kg/m^2^), with locally advanced or metastatic cancer (stages III-IV). Patients were submitted to chemotherapy (6.5%), radical prostatectomy (25.8%), radiation therapy (67.7%), and hormonotherapy (100%). No differences were found between groups at baseline concerning patient sociodemographic and clinic-pathologic characteristics.

**Table 1:** Patient baseline characteristics.

	Usual Care (n=15)	Walking Football (n=16)	*P* value
Age (years)	70.7 ± 6.9	72.8 ± 4.9	0.342
Weight (kg)	81.8 ± 16.3	78.8 ± 10.2	0.539
Height (cm)	168.9 ± 5.8	168.8 ± 6.5	0.977
Body mass index (kg/m^2^)	28.6 ± 5.0	27.9 ± 3.0	0.646
Cancer history			
Disease Stage			0.361
• II	1 (6.7%)	3 (18.8%)	
• III	7 (46.7%)	9 (56.3%)	
• IV	7 (46.7%)	4 (25.0%)	
PSA (ng/mL)	0.6 ± 1.5	1.7 ± 5.9	0.490
Prostatectomy (n, %)	5 (31.3%)	3 (18.8%)	0.354
Orchiectomy (n, %)	0 (0%)	0 (0%)	–
Hormonotherapy (n, %)	15 (100%)	16 (100%)	–
Chemotherapy (n, %)	0 (0%)	2 (12.5%)	0.157
Radiotherapy (n, %)	10 (66.7%)	11 (68.8%)	0.901
Current ADT (n, %)			0.163
• LHRH agonist	12 (80.0%)	14 (87.5%)	
• LHRH agonist and Bicalutamide	2 (13.3%)	0 (0%)	
• LHRH agonist and Enzalutamide	0 (0%)	2 (12.5%)	
• LHRH agonist and Abiraterone	1 (6.7%)	0 (0%)	
ADT time (weeks)	36.1 ± 43.5	23.8 ± 12.2	0.291
Bone Metastases	5 (33.3%)	3 (18.8%)	0.354
Comorbidities			
Diabetes (n, %)	3 (18.8%)	5 (31.3%)	0.414
Hypertension (n, %)	8 (50.0%)	12 (75.0%)	0.144
Hypercholesterolemia (n, %)	8 (50.0%)	8 (50.0%)	1.000
COPD (n, %)	1 (6.7%)	0 (0%)	0.310
Prior CVD (n, %)	1 (6.3%)	2 (12.5%)	0.612
Depression (n, %)	1 (20.0%)	1 (14.3%)	0.906
Smoking (n, %)	1 (6,7%)	1 (12,5%)	1.000

PSA, Prostate Specific Antigen; PCa, Prostate Cancer; ADT, Androgen Deprivation Therapy; LHRH, luteinising hormone-releasing hormone; COPD, Chronic obstructive pulmonary disease; CVD, Cardiovascular Disease.

### Feasibility

Two patients (8%) out of the 25 patients from the WF group withdrew their informed consent before participation, and 2 (11%) discontinued their participation from the 19 patients who initiated the program. The remaining patients (*n*=16) in the WF group attended on average 38 ± 8 training sessions. This corresponded to 81.6 ± 15.9% of the total number of training sessions. The median attendance was 90% (minimum 53% and maximum 98%), with none of the patients completing all the training sessions. Three participants attended less than 70% of the sessions. Moreover, a mean of 13 ± 1 patients attended the sessions, and patients’ level of enjoyment with the training sessions was very high (4.5 ± 0.5 points on the Likert scale).

### Characteristics of the training sessions

Patients showed a mean of 101.9 ± 13.1 bpm during training sessions, which corresponded to 72.8 ± 10.7% of maximum HR. Most of the time of the training sessions was spent on moderate (38.1 ± 16.8 minutes, 45.9 ± 19.4%) and vigorous (22.4 ± 21.5 minutes, 26.8 ± 25.1%) exercise intensity, followed by light (14.7 ± 13.1 minutes, 17.9 ± 15.8%), very light (5.6 ± 10.3 minutes, 6.8 ± 12.2%) and maximum exercise intensities (2.1 ± 6.4 minutes, 2.7 ± 8.4%). The mean perceived exercise effort during the sessions was 13.5 ± 2.6 points on Borg the Scale.

### Quality of life

No differences at baseline were observed in the overall score of health-related QoL (EORTC-QLQ-C30) between the intervention and control groups (*p*=0.883). Moreover, no changes over time were observed in the overall quality of life score in the WF group (median, IQR: 83.3, 66.7-100.0 vs. 83.3, 68.8-100.0 vs. 83.3, 54.2-100.0, *p*=0.462) or the control group (median, IQR: 83.3, 58.3-91.7 vs. 83.3, 66.7-100.0 vs. 83.3, 45.8-100.0, *p*=0.462). No differences were also found for any scale subitem except for diarrhea (**Table 2**). Per-protocol analysis showed no differences in QoL over time when only adherent patients were considered.

**Table 2:** Changes over time in health-related quality of life in walking football and usual care groups.

	Usual Care (*N*=15)				Walking Football (*N*=16)			
	Baseline	8 weeks	16 weeks	*p*-value	Baseline	8 weeks	16 weeks	*p*-value
EORTC QLQ-C30								
**Quality of Life** Global Health Status	83.3 (58.3-91.7)	83.3 (66.7-100.0)	83.3 (45.8-100.0)	0.462	83.3 (66.7-100.0)	83.3 (68.8-100.0)	83.3 (54.2-100.0)	0.674
Functional scales								
Physical functioning	93.3 (73.3-93.3)	86.7 (73.3-93.3)	93.3 (73.3-100.0)	0.167	90.0 (86.7-93.3)	93.3 (86.7-100.0)	93.3 (86.7-100.0)	0.250
Role functioning	100.0 (100.0-100.0)	100.0 (100.0-100.0)	100.0 (100.0-100.0)	0.197	100.0 (100.0-100.0)	100.0 (100.0-100.0)	100.0 (100.0-100.0)	0.336
Emotional functioning	91.7 (75.0-91.7)	91.7 (75.0-100.0)	91.7 (75.0-100.0)	0.384	95.8 (75.0-100.0)	91.7 (75.7-100.0)	95.8 (68.8-100.0)	0.537
Cognitive functioning	83.3 (66.7-100.0)	83.3 (83.3-100.0)	83.3 (83.3-100.0)	0.886	83.3 (83.3-100.0)	100.0 (10.8-100.0)	91.7 (83.3-100.0)	0.478
Social functioning	100.0 (100.0-100.0)	100.0 (100.0-100.0)	100.0 (100.0-100.0)	0.705	100.0 (83.3-100.0)	100.0 (10.8-100.0)	100.0 (100.0-100.0)	0.098
Symptom scales								
Fatigue	22.2 (0.0-33.3)	11.1 (0.0-22.2)	0.0 (0.0-11.1)	0.207	16.7 (0.0-30.6)	11.1 0.0-22.2)	0.0 (0.0-16.7)	0.085
Nausea and Vomiting	0.0 (0.0-0.0)	0.0 (0.0-0.0)	0.0 (0.0-0.0)	0.317	0.0 (0.0-0.0)	0.0 (0.0-0.0)	0.0 (0.0-0.0)	1.000
Pain	16.7 (0.0-16.7)	16.7 (0.0-16.7)	16.7 (0.0-16.7)	0.317	0.0 (0.0-16.7)	0.0 (0.0-16.7)	16.7 (0.0-16.7)	0.132
Dyspnea	0.0 (0.0-0.0)	0.0 (0.0-0.0)	0.0 (0.0-0.0)	0.447	0.0 (0.0-0.0)	0.0 (0.0-0.0)	0.0 (0.0-0.0)	1.000
Insomnia	0.0 (0.0-33.3)	0.0 (0.0-33.3)	0.0 (0.0-33.3)	0.414	0.0 (0.0-33.3)	0.0 (0.0-33.3)	0.0 (0.0-33.3)	0.705
Appetite loss	0.0 (0.0-0.0)	0.0 (0.0-33.3)	0.0 (0.0-33.3)	1.000	0.0 (0.0-0.0)	0.0 (0.0-0.0)	0.0 (0.0-0.0)	0.317
Constipation	0.0 (0.0-33.3)	0.0 (0.0-0.0)	0.0 (0.0-0.0)	0.317	0.0 (0.0-25.0)	0.0 (0.0-33.3)	0.0 (0.0-0.0)	0,257
Diarrhea	0.0 (0.0-0.0)	0.0 (0.0-0.0)	0.0 (0.0-0.0)	0.564	0.0 (0.0-0.0)	0.0 (0.0-33.3)	0.0 (0.0-0.0)	0.046
Financial difficulties	0.0 (0.0-0.0)	0.0 (0.0-0.0)	0.0 (0.0-0.0)	0.807	0.0 (0.0-0.0)	0.0 (0.0-0.0)	0.0 (0.0-0.0)	1.000
EORTC PR25								
Symptoms scales								
Urinary symptoms	87.5 (69.8-95.8)	83.3 (79.2-91.7)	91.7 (75.0-95.8)	0.127	91.7 (87.5-95.8)	89.6 (84.4-95.8)	91.7 (88.5-100.0)	0.927
Incontinence aid	100.0 (100.0-100.0)	100.0 (100.0-100.0)	100.0 (91.7-100.0)	0.317	100.0 (100.0-100.0)	100.0 (100.0-100.0)	100.0 (100.0-100.0)	0.317
Bowel symptoms	100.0 (91.7-100.0)	100.0 (91.7-100.0)	100.0 (91.7-100.0)	0.565	100.0 (91.7-100.0)	100.0 (91.7-100.0)	100.0 (100.0-100.0)	0.042
Hormonal treatment-related	83.3 (72.2-94.4)	83.3 (77.8-94.4)	94.4 (83.3-94.4)	0.132	94.4 (77.8-100.0)	86.1 (79.1-98.6)	91.7 (83.3-100.0)	0.667
Functional scales								
Sexual activity	16.7 (0.0-33.3)	16.7 (0.0-33.3)	66.7 (50.0-100.0)*†	0.000	16.7 (0.0-33.3)	16.7 (0.0-33.3)	83.3 (66.7-100.0) *†	<0.001
Sexual functioning	50.0 (29.1-58.3)	58.3 (25.0-75.0)	70.8 (31.3-87.5)	0.127	58.3 (41.7-75.0)	50.0 (41.7-66.7)	33.3 (25.0-72.9)	0.497

Data is presented as median (25^th^-75^th^ quartiles); *P<0.01 (vs. 8 weeks); † *P*<0.01 (vs. baseline).

### Cardiorespiratory fitness

No differences across treatment groups were observed in CRF at baseline (8.1 ± 1.7 vs. 8.0 ± 1.5 METs *p*=0.865) and between groups over time (-0.1 ± 0.5 vs. 0.3 ± 0.8 METs, *p*=0.147). However, a per-protocol analysis revealed that, when patients who attended less than 70% of the sessions were excluded from the analysis (n=3), there was a significant difference between groups (*p*=0.035), with CRF improving in the WF group from baseline to 16 weeks (8.2 ± 1.6 vs. 8.6 ± 1.5 METs, *p*=0.036) but not in the control group (8.1 ± 1.7 vs. 8.1 ± 1.7 METs, *p*=0.597).

### Muscle strength

No differences were found between groups in handgrip strength and isometric maximal strength in both lower limbs, both at baseline and in the changes over time (**Table 3**). Nonetheless, within-group comparisons showed that handgrip strength and maximal isometric muscle strength in the non-dominant lower limb improved after 8 weeks of WF practice, while no changes over time were observed in the control group.

**Table 3:** Changes over time in muscle strength in walking football and usual care groups.

	Usual Care (*N*=15)				Walking Football (*N*=16)				
	Baseline	8 weeks	16 weeks	Time *p*-value	Baseline	8 weeks	16 weeks	Time *p*-value	Time*Group *p*-value
Muscle Strength									
Handgrip strength, dominant limb (kgf)	29.1 ± 6.4	30.8 ± 4.3	30.9 ± 5.4	0.217	30.7 ± 4.8	36.0 ± 10.5*	32.2 ± 4.6	0.024	0.880
Handgrip strength, non-dominant limb (kgf)	28.0 ± 6.4	29.8 ± 5.2	30.1 ± 5.2	0.146	29.4 ± 4.8	29.6 ± 5.1	30.2 ± 5.8	0.593	0.467
Lower body strength, dominant limb (kgf)	23.4 ± 6.9	22.9 ± 6.4	24.5 ± 8.1	0.581	24.3 ± 4.7	27.2 ± 6.3	26.2 ± 5.7	0.080	0.221
Lower body strength, non-dominant limb (kgf)	23.6 ± 6.8	22.9 ± 6.3	24.6 ± 8.3	0.517	23.9 ± 6.6	26.7 ± 8.0*	27.3 ± 9.1	0.006	0.173
Chair sit-to-stand (number of repetitions)	11.0 ± 2.0	11.9 ± 2.2	11.7 ± 2.9	0.412	13.8 ± 2.9	16.4 ± 3.6**	17.4 ± 4.7**	<0.001	0.035

*Significantly higher than baseline; *p*<0.05; **Significantly higher than baseline; *p*<0.01.

The per-protocol analysis showed no differences between groups in terms of changes over time in both the dominant (*p*=0.94) and non-dominant handgrip strength (*p*=0.37), as well as the dominant (*p*=0.15) and non-dominant leg strength (*p*=0.09). However, the WF group improved maximal isometric leg strength in dominant (24.5 ± 5.1 vs. 28.0 ± 5.6 vs. 27.7 ± 4.9 kgf, *p*=0.006) and non-dominant limbs (24.1 ± 7.2 vs. 27.7 ± 8.3 vs. 28.8 ± 9.5 kgf, *p*=0.001), but not the control group (23.4 ± 6.9 vs. 22.9 ± 6.4 vs. 24.5 ± 8.1 kgf, *p*=0.510; 23.6 ± 6.8 vs. 22.9 ± 6.3 vs. 24.6 ± 8.3 kgf, *p*=0.517).

Moreover, there were significant differences between groups in the number of repetitions completed during the 30-sec chair sit-to-stand test over time (*p*=0.035). While the control group’s performance remained unchanged, the WF group showed improved performance in the 30-sec chair sit-to-stand test (*p*<0.001). Results did not change with per-protocol analysis.

### Balance

Changes in balance over time among WF and control groups are depicted in **Table 4**. There was no significant difference in balance at baseline between the groups. Within-group comparisons showed that balance in the dominant leg improved after 8 weeks and 16 weeks of WF practice (*p*=0.009) but remained unchanged in the control group. No differences were found in the non-dominant leg in both groups. After excluding non-exercise adherent patients (per-protocol analysis) from the walking football group, balance improved significantly after 8 weeks and 16 weeks of intervention in the non-dominant leg (median, IQR: 8.1, 3.3-21.3 vs 17.8, 6.6-38.7 vs 19.2, 7.3-33.5, *p*=0.023) and dominant leg, although with borderline significance (median, IQR: 6.9, 3.2-21.8 vs 16.2, 10.4-33.0 vs 20.1, 10.3-27.0, *p*=0.058).

**Table 4:** Changes over time in balance in walking football and control groups.

	Usual Care (*N*=15)				Walking Football (*N*=16)				
	Baseline	8 weeks	16 weeks	*p*-value	Baseline		8 weeks	16 weeks	*p*-value
Balance									
Dominant limb (sec)	14.2 (5.4-25.0)	14.4 (5.5-22.7)	16.2 (11.6-32.5)	0.262	8.9 (3.2-18.3)		16.3 (10.0-31.7)*	20.3 (10.1-27.6)*	0.009
Non-dominant limb (sec)	23.4 (2.7-32.7)	13.5 (7.5-25.4)	20.0 (3.8-35.2)	0.819	7.9 (3.5-22.4)		17.6 (4.9-34.7)	19.9 (7.0-34.3)	0.099

*Significantly higher than baseline; *p*<0.01.

### Safety

During the WF sessions, 11 patients had a total of 32 AEs. The maximum number of AEs during a single session per patient was 2. Most of the exercise-related events (n=28, 87.5%) occurred during the formal small-sided game setup (7 vs. 7 or 5 vs. 5 games), whereas the remaining 4 events (12.5%) happened during small-sided exercise drills. The majority was related to falls (n=24), which occurred in 10 patients. In most of the falls (n=21, 87.5%), there was no need for the training session interruption; in a small number of falls (n=3,12.5%) there was a momentaneous exercise interruption, but patients resumed the training session thereafter. Moreover, 1 patient reported fatigue on 3 different occasions (9.4%), and 1 patient reported joint pain (n=4, 12.5%), both of which interrupted temporarily the exercise sessions, and resumed after a recovery break. One traumatic injury was registered (hamstrings muscle tear); despite a complete recovery before the end of the intervention, the patient decided to discontinue exercise intervention. Nonetheless, this patient completed all the following assessments and was therefore included in the intention-to-treat analysis.

## Discussion

This study showed that a 16-week program of WF was feasible, safe, and enjoyable. WF practice also significantly improved CRF, muscle strength, and balance in patients with prostate cancer under ADT who adhered to at least 70% of the scheduled exercise sessions. In addition, the results showed that this exercise program allows patients to meet or even overcome the minimal recommendations of physical activity to achieve health benefits (32).

A previous large multicenter study conducted in Denmark also showed that community-based football was a feasible exercise strategy in patients with prostate cancer, by achieving an elevated acceptance rate and retention for 12 weeks and 6 months of the program (33). The current WF program also demonstrated elevated retention. Two patients quit prematurely the program (11%), but compliance was high, as patients attended on average more than 80% of the sessions during 16 weeks. These results are consistent with the elevated level of satisfaction reported. In addition, WF practice was revealed to be safe for patients with prostate cancer, since most of the adverse events related to the exercise program were associated with falls; the great majority of adverse events did not motivate an interruption of the session, and when occurring patients resumed the training session. Only one major traumatic injury (muscle tear) was reported, motivating a permanent interruption of the intervention.

We also observed significant improvements in CRF, muscle strength, and balance in patients who were enrolled in the WF program and complied with at least 70% of the WF sessions. These results are especially relevant because cancer treatments, particularly ADT, can present an overall important burden, eliciting a negative impact on muscle mass and strength, CRF, functional decline, and fatigue (34, 35). It has been shown that prolonged ADT exposure is associated with reduced CRF and increased cardiovascular mortality in patients with prostate cancer (36). Also, muscle loss during hormone treatment is independently associated with increased non-cancer mortality (37). These data reinforce the potential relevance of improvements in physical fitness in prostate cancer patients under ADT. There is evidence showing that aerobic and resistance training can promote significant improvements in fat mass, lean mass, muscle strength, functional capacity, and CRF in patients with prostate cancer during and after treatment (38, 39). Our results add to the current evidence by suggesting that a WF program is an effective exercise strategy to increase physical fitness in patients with prostate cancer. It also shows that WF practice may promote improvements in balance. Notably, current and past patients under ADT are more than twice as likely to have fallen, whilst also presenting more recurrent falling and fall-related injuries compared to men who were never exposed; they are also more likely to be classified as pre-fail than non-users of ADT (40). A recent meta-analysis also concluded that the use of androgen receptor inhibitors is associated with an increased risk of falls and fractures in patients with prostate cancer (41). Even though this was not measured directly, the improvements observed in balance in the WF group suggest that WF practice may be an effective approach to prevent falls and fractures, particularly as most of our patients were older adults.

Previous meta-analyses including randomized clinical trials have shown that exercise training improves QoL in patients with prostate cancer under ADT (42, 43). A recent meta-analysis of 18 randomized controlled trials, including 1477 patients with prostate cancer undergoing androgen deprivation therapy, reported that supervised exercise therapy has a moderately positive effect on disease-specific quality of life compared to no exercise therapy (44). On the other hand, another recent meta-analysis comprising 17 randomized controlled trials, involving 1361 patients with prostate cancer who had received cancer treatment, concluded that exercise had a small effect on cancer-specific QoL, and no differences were observed between exercise modalities (45). In addition, like a previous report (33), we did not observe changes in health-related QoL in patients with prostate cancer that participated in WF practice. Differences in age, assessment methods, treatment regimens, and training programs may explain, at least in part, the discrepancies in results. Of mention, in the current study, patients reported relatively high values of overall QoL at baseline compared to the reference values (46), which may have potentially decreased the margin of improvement in wellbeing with the exercise training.

### Limitations

The main limitation of this study is the greater-than-expected loss of patients (38%) after randomization. Although a few patients withdrew (n=3, 6%) their informed consent after being allocated to one of the two groups, and 6 (12%) patients were lost to follow-up, most patients (n=10, 20%) were not enrolled in the trial due to positive exercise tests. Despite this might have resulted in some loss of power, this well-controlled feasibility study highlights the importance of the baseline clinical assessment to determine the safety of exercise training programs in cancer patients, especially in older patients with prostate cancer under androgen deprivation therapy and with multiple cardiovascular comorbidities and cardiovascular risk factors. Osteoporosis is a possible consequence of hormonal therapy. However, our findings in terms of safety cannot be generalized to patients with osteoporosis as they were excluded from this study. Exercise training targeting the musculoskeletal system, involving impact loading exercises plus resistance training, has been shown to attenuate the decline in the spine and femoral neck bone mineral density in patients with prostate cancer (47). Walking football may also be an effective strategy to mitigate the adverse effects of hormonal therapy on bone health, but future studies must address the balance between the risks and benefits of this mode of exercise in this specific population.

## Conclusions

This study suggests that WF is a safe, enjoyable, and feasible strategy to meet physical activity recommendations in patients with prostate cancer under hormonal therapy. In addition, cardiorespiratory fitness, muscle strength, and balance are likely to improve in patients who show good adherence to WF.

## Data availability statement

The datasets presented in this article are not readily available because Dataset will be available for researchers who provide a methodologically sound proposal. Requests to access the datasets should be directed to AJA, ajalves@umaia.pt.

## Ethics statement

The studies involving human participants were reviewed and approved by Ethics committee of Vila Nova de Gaia-Espinho Hospital Centre. The patients/participants provided their written informed consent to participate in this study.

## Author contributions

AC, PA, EV, AJ, SV, JB, and AJA conceptualized the trial and were responsible for designing the study and the plan for analysis. AC, SC, PA, EV, AJ, SV, JB, and AJA led the implementation of the study design. AC, SC, RA, AA, JS, AJ, and SV were responsible for patient recruitment and clinical evaluation. AC, PA, CC, CG, SC, RA, TC, EV, MT, AA, JS, AJ, SV, JB, and AJA were responsible for collecting data, monitoring participants, and supervising the exercise intervention. The manuscript was written, read, and edited by all authors. All authors contributed to the article and approved the submitted version.
